# Supplementary material for: A clathrin coat assembly role for the muniscin protein central linker revealed by TALEN-mediated gene editing
Source: eLife. 2014 Oct 10;3:e04137. doi: 10.7554/eLife.04137 (PMC4215538; doi:10.7554/eLife.04137)
Supplement: Supplementary file 1. — List of various constructs used in this study and the sets of specific primers, restriction sites, plasmids and the methods of cloning used to design these constructs. DOI: http://dx.doi.org/10.7554/eLife.04137.024 [file elife04137s001.doc]

| Gene product/Protein | Plasmid | Oligonucleotide sequence | Restriction sites | Notes |
| --- | --- | --- | --- | --- |
| GST-FCHO1 (316-467) | pGEX-4T-1-His_10_ | 5' CCG CGT GGA TCC CCG GAA TTC ACA TGT CCA GAG GTG GAT GAA 3'  5'GTG CGG TCG ACC CGG GAA TTC CGA GGA GGA GAA AGG GGA GGG 3' | EcoRI | Cold fusion cloning |
| GST-FCHO2 (314-444) | pGEX-4T-1-His_10_ | 5' CCG CGT GGA TCC CCG GAA TTC AAC ATT CCT GAT GTA GAT GAA GAA 3'  5'GTG CGG TCG ACC CGG GAA TTC AGT TAG TGA AGA TGA AGA AGA TGA 3' | EcoRI | Cold fusion cloning |
| GST-Sgip1 (77-214) | pGEX-4T-1-His_10_ | 5'CCG CGT GGA TCC CCG GAA TTC AAC TCA CCT GAG CTA GAT GAA 3'  5'GTG CGG TCG ACC CGG GAA TTC CGT CTT CTG CTC ATC AAA AGC 3' | EcoRI | Cold fusion cloning |
| GST-EPS15 (595-896) | pGEX-4T-1-His_10_ | 5' CTG AAT TCT CAA AAG AGG AAG ATC CAT TTA ATG 3'  5' CCT CTC GAG TCA TGC TTC TGA TAT CTC AGA TTT G 3' | EcoRI/XhoI | Directional cloning |
| GST-EPS15 (595-896) ∆617-636 | pGEX-4T-1-His_10_ | 5'-/5Phos/GGA AAA ATC GAT CCA TTT GGT GGT G 3'  5'-/5Phos/CAA GTT TGT ATC TGC AAC TGG ACC 3' | EcoRI/XhoI | Phusion PCR |
| GST-EPS15 (595-740) | pGEX-4T-1 | 5' GAA GAT CCT TTT CGT TGA GCC ACA TCG AGC TCT G 3'  5' CAG AGC TCG ATG TGG CTC AAC GAA AAG GAT CTT C 3' | EcoRI/XhoI | QuikChange  mutagenesis |
| GFP-FCHO1 (1-889) | pEGFP-C1 | 5' ATT GAA TTC TAT GTC GTA TTT TGG CGA GC 3'  5' GCG TCG ACT CAG CAG CTC ACC AGG TAC 3' | EcoRI/SalI | Directional cloning |
| GFP-FCHO1 (1-609) | pEGFP-C1 | 5' CAG ACA GGA CAC TGA GTC TCC CGG G 3'  5' CCC GGG AGA CTC AGT GTC CTG TCT G 3' | EcoRI/SalI | QuikChange  mutagenesis |
| GFP-FCHO1 (1-467) | pEGFP-C1 | 5' CCC TTT CTC CTC CTC GTA GCC CGA AAA CGT GG 3'  5' CCA CGT TTT CGG GCT ACG AGG AGG AGA AAG GG 3' | EcoRI/SalI | QuikChange mutagenesis |
| GFP-FCHO1 (1-416) | pEGFP-C1 | 5' CGG TCT GCC CCC TGA ACC AGC AGC TGT G 3'  5' CAC AGC TGC TGG TTC AGG GGG CAG ACC G 3' | EcoRI/SalI | QuikChange mutagenesis |
| GFP-FCHO1 (1-370) | pEGFP-C1 | 5' CCG GGC TCC AGC CTG AAG CCC CGA GGC AG 3'  5' CTG CCT CGG GGC TTC AGG CTG GAG CCC GG 3' | EcoRI/SalI | QuikChange mutagenesis |
| GFP-FCHO1 (1-316) | pEGFP-C1 | 5' CCC GAT TCA GGG ACA TGA CCA GAG GTG GAT G 3'  5' CAT CCA CCT CTG GTC ATG TCC CTG AAT CGG G 3' | EcoRI/SalI | QuikChange mutagenesis |
| GFP-FCHO1 (1-275) | pEGFP-C1 | 5' CAG TGC GGC TGC CTA GCA GGA AGC GAT GAA ACG 3'  5' CGT TTC ATC GCT TCC TGC TAG GCA GCC GCA CTG 3' | EcoRI/SalI | QuikChange mutagenesis |
| GFP-FCHO1 (265-889) | pEGFP-C1 | 5' ATA GAA TTC ACC TCT GGA CTT CGA GGC 3'  5' GCG TCG ACT CAG CAG CTC ACC AGG TAC 3' | EcoRI/SalI | Directional cloning |
| GFP-FCHO1 (265-609) | pEGFP-C1 | 5' CAG ACA GGA CAC TGA GTC TCC CGG G 3'  5' CCC GGG AGA CTC AGT GTC CTG TCT G 3' | EcoRI/SalI | QuikChange mutagenesis |
| GFP-FCHO1 (609-889) | pEGFP-C1 | 5' ATA GAA TTC ACA CGG AGT CTC CCG GG 3'  5' GCG TCG ACT CAG CAG CTC ACC AGG TAC 3' | EcoRI/SalI | Directional cloning |
| GFP-FCHO1 (1-889; ∆316-339) | pEGFP-C1 | 5'-/5Phos/TCC CGT TTC TCG TCC AGC GAC TCC 3'  5'-/5Phos/CCC TGA ATC GGG CTC CAG GAA ATC 3' | EcoRI/SalI | Phusion PCR |
| GFP-FCHO1(1-889; ∆316-467) | pEGFP-C1 | 5'-/5Phos/TCG CCC GAA AAC GTG GAG GAT TCC  5'-/5Phos/CCC TGA ATC GGG CTC CAG GAA ATC 3' | EcoRI/SalI | Phusion PCR |
| GFP-FCHO2 (1-810) | pEGFP-C1 | 5' GCT TCG AAT TCT GCA GTC GAC ATG GTC ATG GCG TAT TTC GTC 3'  5' CGG GCC CGC GGT ACC GTC GAC GCT AAA TTA CTT TGT GCT TGC TCT TC 3' | SalI | Cold fusion cloning |
| GFP-Sgip1 (1-806) | pEGFP-C1 | 5' TAC AAG TCC GGA CTC AGA TCT ATG ATG GAA GGA CTG AAA AA 3'  5' CGA AGC TTG AGC TCG AGA TCT TTA GTT ATC TGC CAA GTA CT 3' | BglII | Cold fusion cloning |
| GFP-Sgip1 (1-514) | pEGFP-C1 | 5' CAA CTA ACT CAC TGA GCT AAG CCA CCA CTC CCA CAG TT 3'  5' AAC TGT GGG AGT GGT GGC TTA GCT CAG TGA GTT AGT TG 3' | BglII | QuikChange mutagenesis |
| Tac-FCHO1 linker (265-609) | pcDNA3.1 | 5' CAG ACA GGA CAC TGA GTC TCC CGG G 3'  5' CCC GGG AGA CTC AGT GTC CTG TCT G 3' | EcoRV/NotI | QuikChange mutagenesis |
| Tac-FCHO1 µHD (609-889) | pcDNA3.1 | 5' ATG ATA TCC ACG GAG TCT CCC GGG G 3'  5' TAG CGG CCG CTC AGC AGC TCA CCA GGT ACA TCC 3' | EcoRV/NotI | Directional cloning |
| Tac-FCHO1 linker + µHD (265-889) | pcDNA3.1 | 5' ATG ATA TCC CTC TGG ACT TCG AGG CAT ACA G 3'  5' TAG CGG CCG CTC AGC AGC TCA CCA GGT ACA TCC 3' | EcoRV/NotI | Directional cloning |

**Supplementary Table**- List of various constructs used in this study and the sets of specific primers, restriction sites, plasmids and the methods of cloning used to design these constructs.
